# Hybrid Energy Storage of Ni(OH)_2_-coated N-doped Graphene Aerogel//N-doped Graphene Aerogel for the Replacement of NiCd and NiMH Batteries

**DOI:** 10.1038/s41598-017-01191-8

**Published:** 2017-04-25

**Authors:** Pichamon Sirisinudomkit, Pawin Iamprasertkun, Atiweena Krittayavathananon, Tanut Pettong, Peerapan Dittanet, Montree Sawangphruk

**Affiliations:** 1 Department of Chemical and Biomolecular Engineering, School of Energy Science and Engineering, Vidyasirimedhi Institute of Science and Technology, Rayong, 21210 Thailand; 2 Department of Chemical Engineering, Centre for Advanced Studies in Nanotechnology and Its Applications in Chemical Food and Agricultural Industries, and NANOTEC-KU-Centre of Excellence on Nanoscale Materials Design for Green Nanotechnology, Kasetsart University, Bangkok, 10900 Thailand

## Abstract

Although Nickel–Cadmium (NiCd) and Nickel–metal hydride (NiMH) batteries have been widely used, their drawbacks including toxic Cd and expensive La alloy at the negative electrodes, low energy density (40–60 Wh/kg for NiCd and 140–300 Wh/L for NiMH), low power density (150 W/kg for NiCd and 1000 W/kg for NiMH), and low working potential (1.2 V) limit their applications. In this work, Cd and La alloy were replaced with N-doped reduced graphene oxide aerogel (N-rGO_ae_) providing a hybrid energy storage (HES) having the battery and supercapacitor effects. The HES of Ni(OH)_2_-coated N-rGO_ae_//N-rGO_ae_ provides 1.5 V, a specific energy of 146 Wh/kg, a maximum specific power of 7705 W/kg, and high capacity retention over 84.6% after 5000 cycles. The mass change at the positive electrode during charging/discharging is 8.5 µg cm^−2^ owing to the insertion/desertion of solvated OH^−^ into the α-Ni(OH)_2_-coated N-rGO_ae_. At the negative electrode, the mass change of the solvated K^+^, physically adsorbed/desorbed to the N-rGO_ae_, is 7.5 μg cm^−2^. *In situ* X-ray absorption spectroscopy (XAS) shows highly reversible redox reaction of α-Ni(OH)_2_. The as-fabricated device without using toxic Cd and expensive La alloy has a potential as a candidate of NiCd and NiMH.

## Introduction

Although the Nickel–Cadmium (NiCd) and Nickel–metal hydride (NiMH) batteries have been widely used, their drawbacks including toxic cadmium and expensive lanthanum alloy at the negative electrodes, low energy density (40–60 Wh/kg for NiCd and 140–300 Wh/L for NiMH), low power density (150 W/kg for NiCd and 1000 W/kg for NiMH), and low working potential (1.2 V) limit their applications in electric vehicles, laptops, and mobile phones when compared with Li-ion batteries^1, 2^. At the positive electrode, NiOOH and Ni(OH)_2_ are used for NiCd and NiMH, respectively.

Recently Ni(OH)_2_-based supercapacitors have also attached a superior attention because of its high theoretical specific capacitance (2,082 F g^−1^)^3, 4^, affordable price, abundant natural resources, and excellent environment compatibility^4^. More interestingly, some pervious work even reported the remarkably high specific capacitances of Ni(OH)_2_-based supercapacitors, which are over its theoretical value^5–10^. Generally, α-Ni(OH)_2_ and β-Ni(OH)_2_ are the two common phases of Ni(OH)_2_, which have been using as the supercapacitor electrodes^11^. α-Ni(OH)_2_/γ-NiOOH redox couple can transform to β-Ni(OH)_2_/β-NiOOH redox couple easily, which can lead to a specific capacitance decay^12, 13^. However, many β-Ni(OH)_2_-based supercapacitors with great electrochemical performance have still been reported^14–17^. As the results, it needs more understanding in the charge storage mechanism of Ni(OH)_2_-based supercapacitors. It is also necessary to note here that there is a critic arguing that Ni(OH)_2_ storing the charges via a phase transformation cannot be considered as the conventional supercapacitor materials^18, 19^. Our results here also show that Ni(OH)_2_-coated N-doped reduced graphene aerogel (N-rGO_ae_) mainly stores the electronic charge via a bulk redox reaction especially as very slow charging/discharging rates.

Although the reaction mechanism of the Ni(OH)_2_ itself follows the reversible reaction (1)^20, 21^, the charge storage mechanism of Ni(OH)_2_ composites e.g., Ni(OH)_2_/graphene is not yet clear.

1 $$\alpha  \mbox{-} \mathrm{Ni}{({\rm{OH}})}_{2}+{{\rm{OH}}}^{-}\leftrightarrow \gamma  \mbox{-} \mathrm{NiOOH}+{{\rm{H}}}_{2}{\rm{O}}+{{\rm{e}}}^{-}$$

Note, the reaction (1) originally introduced by Bode *et al*.^1, 2^ *is well known in NiCd and NiMH batteries*. For the Ni(OH)_2_ supercapacitors, the theoretical specific capacitance of Ni(OH)_2_ is based on the reaction (1). However, the electrochemical property of α-Ni(OH)_2_ is still far lower than its theoretical value due to its insulating property, poor capacity retention and irreversible reaction^22^. To overcome the drawbacks of the Ni(OH)_2_, it was produced on the conducting graphene sheets providing a specific energy of ca. 37 W h kg^−1^ ^3^. In this research, α-Ni(OH)_2_ is coated on N-rGO_ae_ and used for the asymmetric energy storage (AES) of Ni(OH)_2-_N-rGO_ae_//N-rGO_ae_ leading to high specific energy and power. This is because N-rGO_ae_ has high electrical conductivity (2 × 10^3^ S cm^−1^)^23^, high ionic conductivity due to nitrogen- and oxygen-containing groups and high porosity, which is good for the diffusion of the electrolyte^24, 25^. The as-fabricated AES of the Ni(OH)_2_-coated N-rGO_ae_//N-rGO_ae_ in a CR2016 coin cell can deliver a wide cell working potential up to 1.5 V and high specific energy and maximum specific power of 146 Wh kg^−1^ and 7705 W kg^−1^, respectively.

In addition, the charge storage mechanisms of both α-Ni(OH)_2_-coated N-rGO_ae_ and N-rGO_ae_ are investigated in this work by means of *in situ* X-ray absorption spectroscopy (XAS) and *in situ* electrochemical quartz crystal microbalance (EQCM). The result shows that the AES of α-Ni(OH)_2_-coated N-rGO_ae_//N-rGO_ae_ can store charges via both physical adsorption and redox reaction.

## Results and Discussion

### Morphologies of the as-synthesised materials

**Figure 1:**
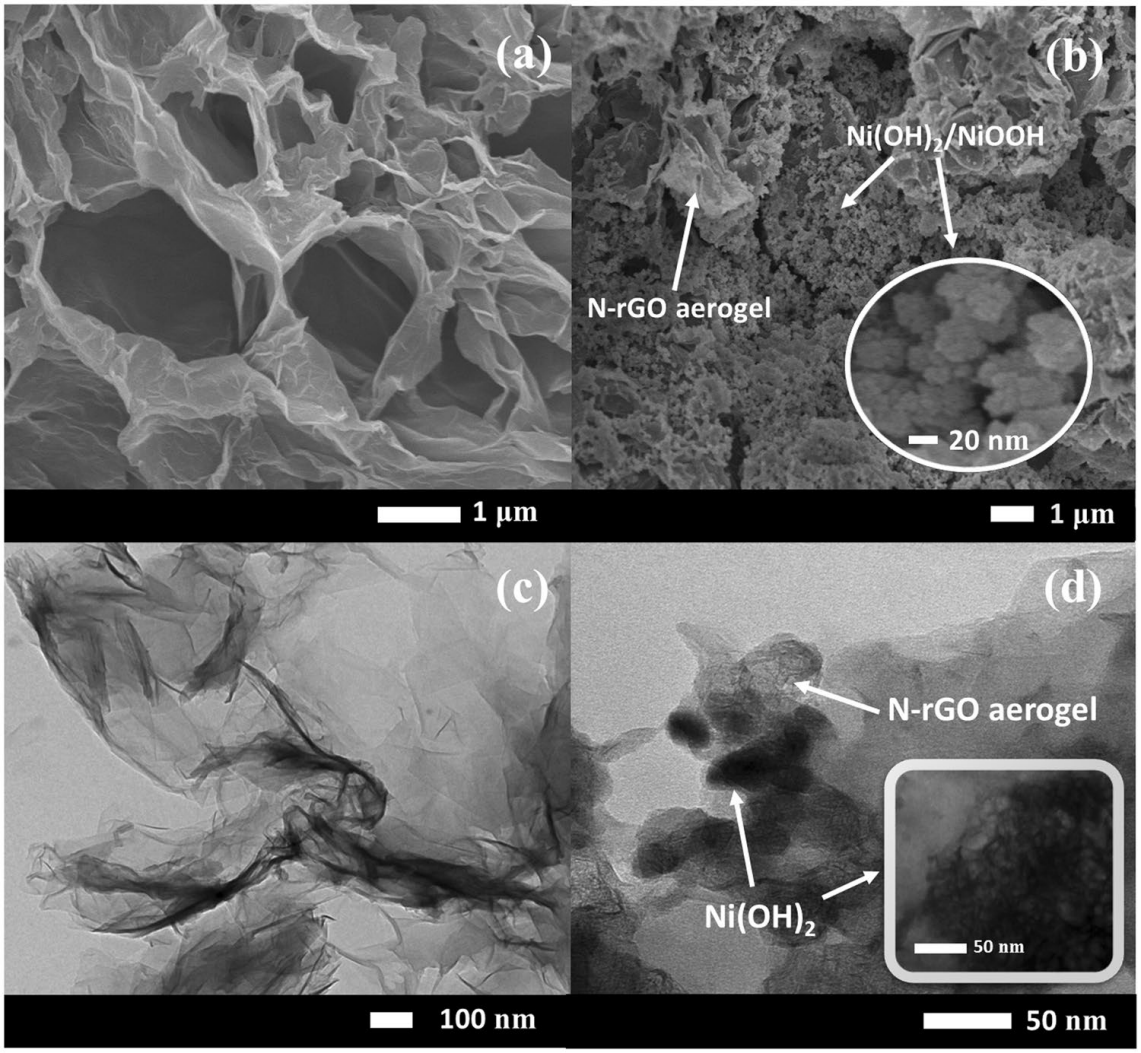
FE-SEM images of (**a**) N-rGO_ae_ spray-coated on CFP, (**b**) Ni(OH)_2_ electrodeposited on the N-rGO_ae_/CFP. TEM images of (**c**) N-rGO_ae_ and (**d**) Ni(OH)_2_ coated on N-rGO_ae_.

The morphology of the as-synthesized materials was investigated by Field Emission Scanning Electron Microscopy (FE-SEM). The 3D porous network structure of N-rGO_ae_ was coated on flexible carbon fiber paper (CFP) substrate (see Fig. 1a) for which the N-rGO sheets are interconnected each other forming high porosity aerogel. Other physicochemical properties of the N-rGO_ae_ were previously reported by our group elsewhere^26, 27^. An FE-SEM image of Ni(OH)_2_ electrodeposited on the N-rGO_ae_/CFP shows that Ni(OH)_2_ was fully loaded to the pores of the N-rGO_ae_ and the Ni(OH)_2_ nanoparticles were subsequently coated on the top layer in Fig. 1b. Note, lower magnification FE-SEM images of CFP and the as-electrodeposited α-Ni(OH)_2_ on CFP are displayed in Figure S1 and b, respectively. In addition, a *transmission electron microscopy* (TEM) image of N-rGO_ae_ in Fig. 1c indicates a few layers of N-rGO_ae_ sheets forming the wrinkle structure confirming the framework structure of the aerogel. This structure can address the restacking issue typically found in 2D graphene or rGO nanosheets. The interconnected porous structure can also deliver large surface area and porosity as well as great electrical conductivity. A TEM image of Ni(OH)_2_-coated N-rGO_ae_ sheets in Fig. 1d displays the dark Ni(OH)_2_ particles with 25 nm in diameter dispersed on the N-rGO_ae_ layers. Note, XRD, Raman, and XPS of Ni(OH)_2_, N-rGO_ae_ and Ni(OH)_2_-N-rGO_ae_ are shown in Figures S2 and S3 of the Supporting Information.

### Charge storage mechanisms

**Figure 2:**
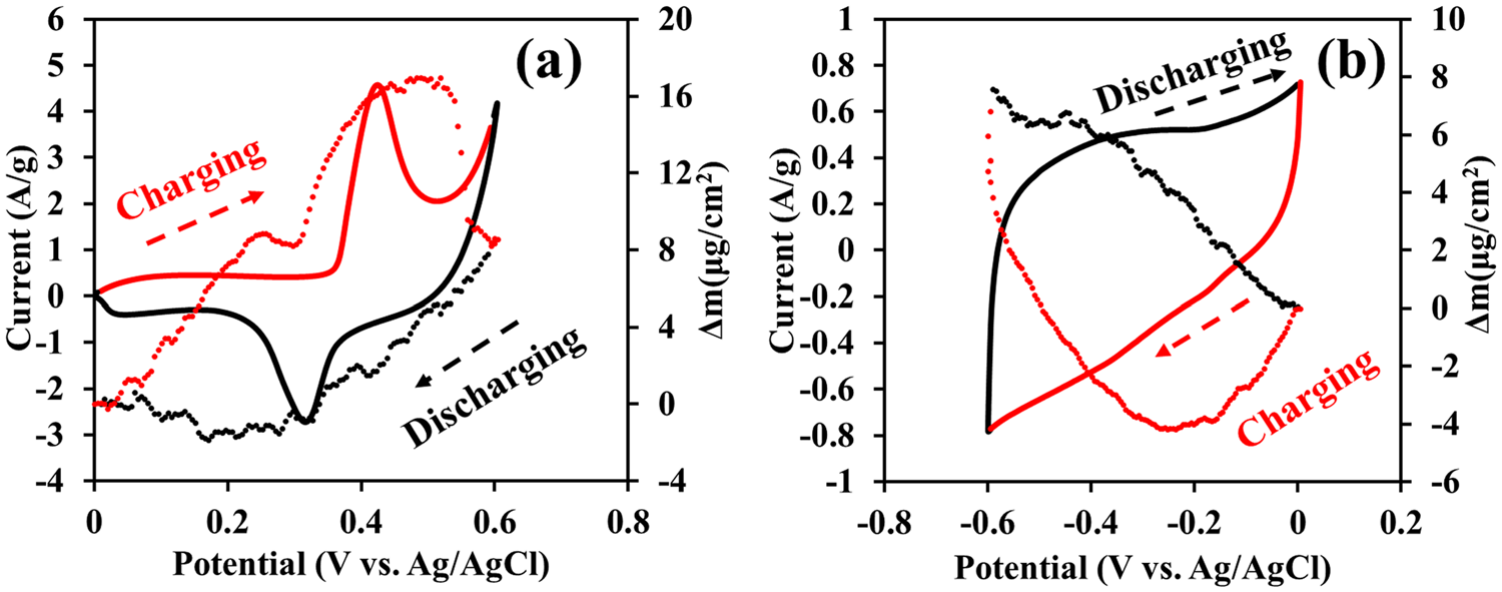
EQCM responses and CVs of (**a**) α-Ni(OH)_2_-N-rGO_ae_ and (**b**) N-rGO_ae_ at 10 mVs^−1^. Note, dash lines present the related mass changes of the electrodes during charging/discharging.

The charge-discharge mechanisms of the α-Ni(OH)_2_-N-rGO_ae_ and N-rGO_ae_ electrodes were investigated using the EQCM together with the CV technique. The CV of the α-Ni(OH)_2_-coated N-rGO_ae_ electrode is presented in Fig. 2a. The forward scan started from 0 to 0.6 V vs. Ag/AgCl for which an anodic peak occurs at 0.43 V vs. Ag/AgCl indicating that α-Ni(OH)_2_ reacts with OH^−^ providing *γ*-NiOOH, a water molecule, and an electron through the oxidation^13–15^. Along this forward scan, the mass change (Δ*m*) of the electrode is about 8.5 µg cm^−2^ owing to the insertion of solvated OH^−^ ions into α-Ni(OH)_2_ interlayer separation. Also, the increasing Δ*m* during the forward scan refers to the charging mechanism^28^. On the other hand, the backward scan (the discharging process) was swept back from 0.6 to 0 V vs. Ag/AgCl. The Δ*m* of the electrode is reduced back to an original value, 0 μg cm^−2^ (see a black dash line in Fig. 2a) due to the full desertion of solvated OH^−^. It can be concluded from the EQCM/CV results that the charge storage mechanism of the α-Ni(OH)_2_-N-rGO_ae_ is based on the interaction chemistry especially at slow scan rates. α-Ni(OH)_2_-N-rGO_ae_ is then considered as the battery-type electrode material.

For the N-rGO_ae_ electrode, the CV and Δ*m* vs. the applied potentials are shown in Fig. 2b. The CV with a working voltage of ca. 0.6 V exhibits a most likely rectangular shape representing a good EDLC behavior of the N-rGO_ae_. Broad peaks also represent the redox reactions of N-containing groups^26, 27^. For the charging process, the range of potential from 0 V to −0.6 V vs. Ag/AgCl was applied for which the Δ*m* is decreased at a working window from 0 to −0.2 V vs. Ag/AgCl due to the desorption of ionic species initially adsorbed/absorbed when the electrode is immersed in the electrolytes without applying potential. This counter-ion desorption observed in this work is in good agreement with the new perspective charge storage mechanism recently proposed using *in situ* NMR^29^. At a potential range between −0.2 V and −0.6 V vs. Ag/AgCl, the Δ*m* is rapidly increased to 7.5 μg cm^−2^ due to the adsorption/absorption of hydrated K^+^ within the active pores of N-rGO_ae_.

To confirm the charge storage behavior of the as-prepared α-Ni(OH)_2_-N-rGO_ae_ electrode, the *in situ* XAS technique was performed with the chronoamperometry in 1 M KOH electrolyte during applying the potentials stepped from 0.0, 0.35, 0.5 and 0.6 V vs. Ag/AgCl and the following backward scan at the applied potentials from 0.6, 0.4, 0.18 to 0 V vs. Ag/AgCl. Note, the Ni K-edge XANES of the samples were recorded and compared with Ni foil (Ni^0^, 8333.00 eV), NiO (Ni^2+^, 8345.20 eV) and LaNiO_3_ (Ni^3+^, 8348.20 eV) standards^30^.

**Figure 3:**
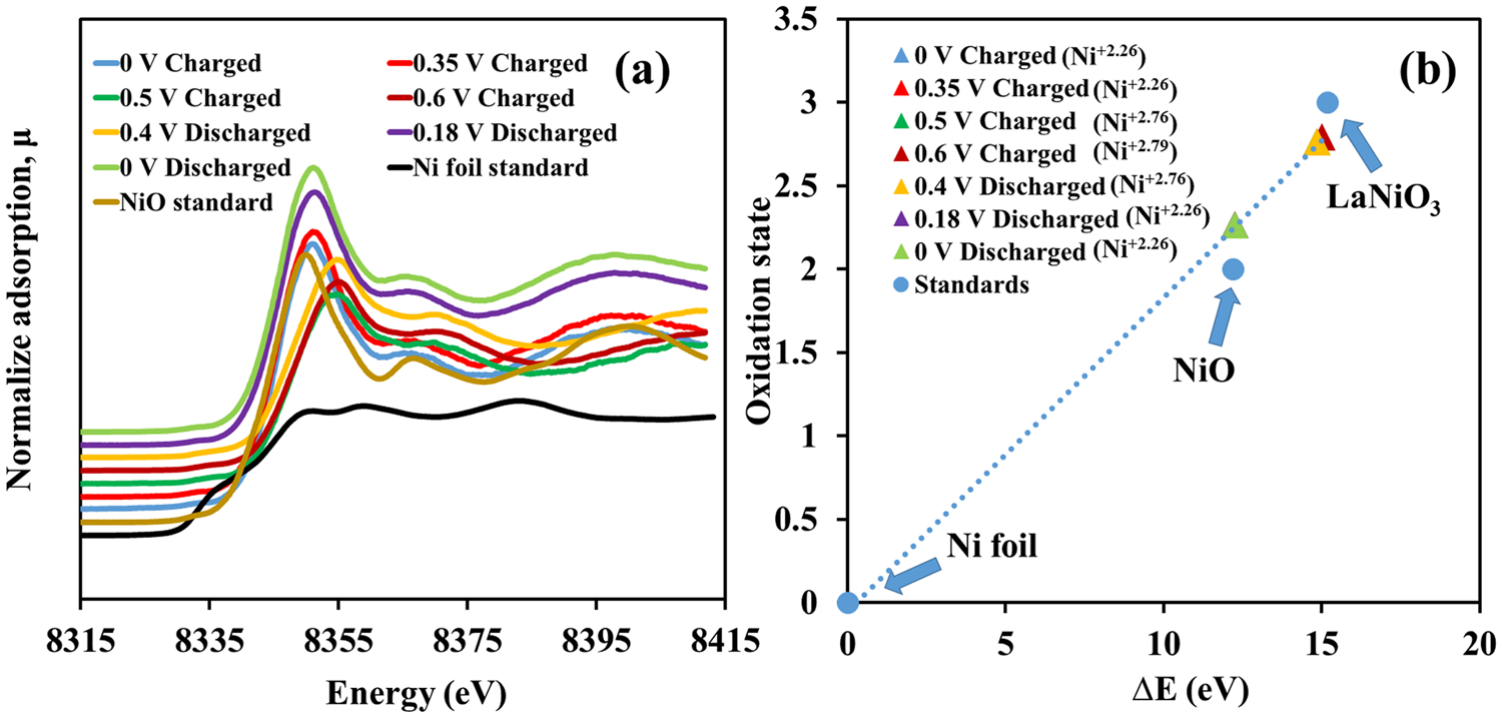
(**a**) *In situ* high-resolution Ni K-edge fluorescence XAS spectra of the as-prepared Ni(OH)_2_-N-rGO_ae_ electrode and Ni standard compounds and (**b**) the oxidation states vs. ΔE (eV) of the Ni(OH)_2_-N-rGO_ae_ electrode during charging/discharging by a chronoamperometry method at different applied potentials vs. Ag/AgCl.

In Fig. 3a and b, the Ni K-edge fluorescence energy of the Ni(OH)_2_-N-rGO_ae_ electrode charged at 0.0 V vs. Ag/AgCl is 8345.25 eV (Ni^+2.26^) and the adsorption energy remains constant at +2.26 until 0.35 V vs. Ag/AgCl before increasing to the energy value of 8347.84 (Ni^+2.76^) and 8348.00 eV (Ni^+2.79^) when the charging potentials were applied to 0.5 and 0.6 V vs. Ag/AgCl, respectively. This is due to the oxidation reaction of Ni(OH)_2_ with OH^−^ providing NiOOH. This result further confirms the EQCM results. For the discharging process, the Ni K-edge fluorescence energy of the Ni(OH)_2_-N-rGO_ae_ electrode discharged at 0.4 V vs. Ag/AgCl is 8347.84 eV (Ni^+2.76^) and continues to stable at 8345.25 eV (Ni^+2.26^) when discharged at 0.18 V and 0 V vs. Ag/AgCl. These results also confirm the reversible redox reaction of Ni(OH)_2_-N-rGO_ae_ electrode.

### Hybrid energy storage of Ni(OH)_2_-N-rGO_ae_//N-rGO_ae_

**Figure 4:**
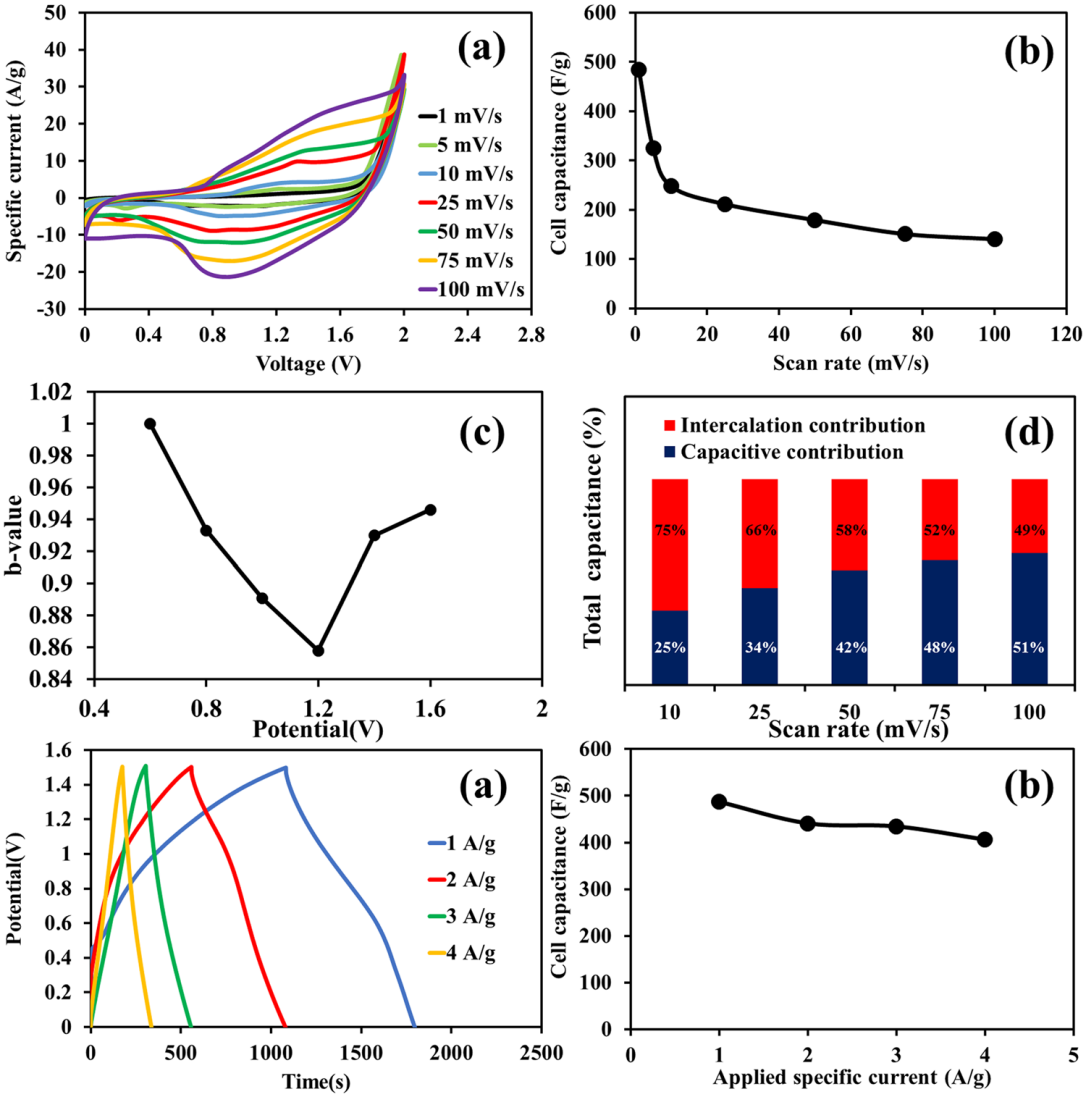
(**a**) CV curves at different scan rates, (**b**) specific capacitance vs. scan rate, (**c**) *b*-value vs. potential (V), (**d**) total capacitances at different scan rates, (**e**) GCD curves and (**f**) specific capacitance vs. applied currents of the Ni(OH)_2_-N-rGO_ae_//N-rGO_ae_ AES. Note, the total active material of the device is 4.0 mg consisting of 1.47 mg of Ni(OH)_2_-N-rGO_ae_ at the positive electrode and 2.53 mg of N-rGO_ae_ at the negative electrode.

The as-fabricated Ni(OH)_2_-N-rGO_ae_//N-rGO_ae_ AES can provide a wide working potential (See Fig. 4a), which is greater than all aqueous-based Ni(OH)_2_ symmetric supercapacitors^3, 9, 31^. Note, the total active material of the device is 4.0 mg consisting of 1.47 mg of Ni(OH)_2_-N-rGO_ae_ at the positive electrode and 2.53 mg of N-rGO_ae_ at the negative electrode. In addition, the CV shows the characteristics of the pseudocapacitors consisting of both capacitive and faradaic currents. The couple redox peaks are observed owing to the intercalation and deintercalation of the solvated OH^−^(H_2_O)_*n*_ (*where n* ≥ 1)^32^. Whilst, the capacitive current observed in the CVs is mostly due to the adsorption of hydrated K^+^ on the N-rGO_ae_ negative electrode surface. The cell specific capacitances of the AES device are 484.3, 324.4, 247.8, 211.4, 179.1, 150.8 and 140.4 F g^−1^ at 1, 5, 10, 25, 50, 75 and 100 mV s^−1^, respectively (Fig. 4b). From a power law, *b* value was then calculated^33^, *i* = *av* ^*b*^ where the current is represented as *i* and the scan rate is represented as *v*. The adjustable parameters are expressed as *a* and *b*. Typically, the *b* value is equal to 1.0 for non-diffusion-controlled surface capacitive (an EDLC-like electrochemical feature) and equal to 0.5 for diffusion-controlled redox reaction, which is a typical battery behavior^19, 34^. From the beginning, the *b* value of the cell device is almost amounted to 1 before dropping down to 0.86 (see Fig. 4c) indicating that the specific capacitances of the device are contributed from both diffusion-controlled battery-type behavior and capacitive effect. The capacitive contribution fraction at different scan rates can be examined from *i*(*V*) = *k*_1_v + *k*_2_ *v* ^0.5^ where *k*_*1*_ and *k*_*2*_ are alterable parameters determined from the slope and y-axis intercept of the plots between *i*(*V*) and *v*, respectively. The capacitive and intercalation contribution fractions are shown in Fig. 4d. It is clearly observed that they are sensitive to scan rates. In addition to the CV results, GCD curves were carried out and shown in Fig. 4e for which the shapes of the charge and discharge curves imply good conductivity, excellent coulombic efficiency and high electrochemical reversibility of the AES. Besides, the calculated cell capacitances are 487.1, 441.1, 434.5 and 406.5 F g^−1^ at 1, 2, 3 and 4 A g^−1^, respectively (see Fig. 4f). The actual working potential window excluding the *iR* drop is ca. 1.5 V. When compared with other previous report, the as-fabricated Ni(OH)_2_-N-rGO_ae_//N-rGO_ae_ AES exhibits significantly higher specific capacitance than other Ni(OH)_2_-based asymmetric supercapacitors (see Table S1 of the Supporting Information).

**Figure 5:**
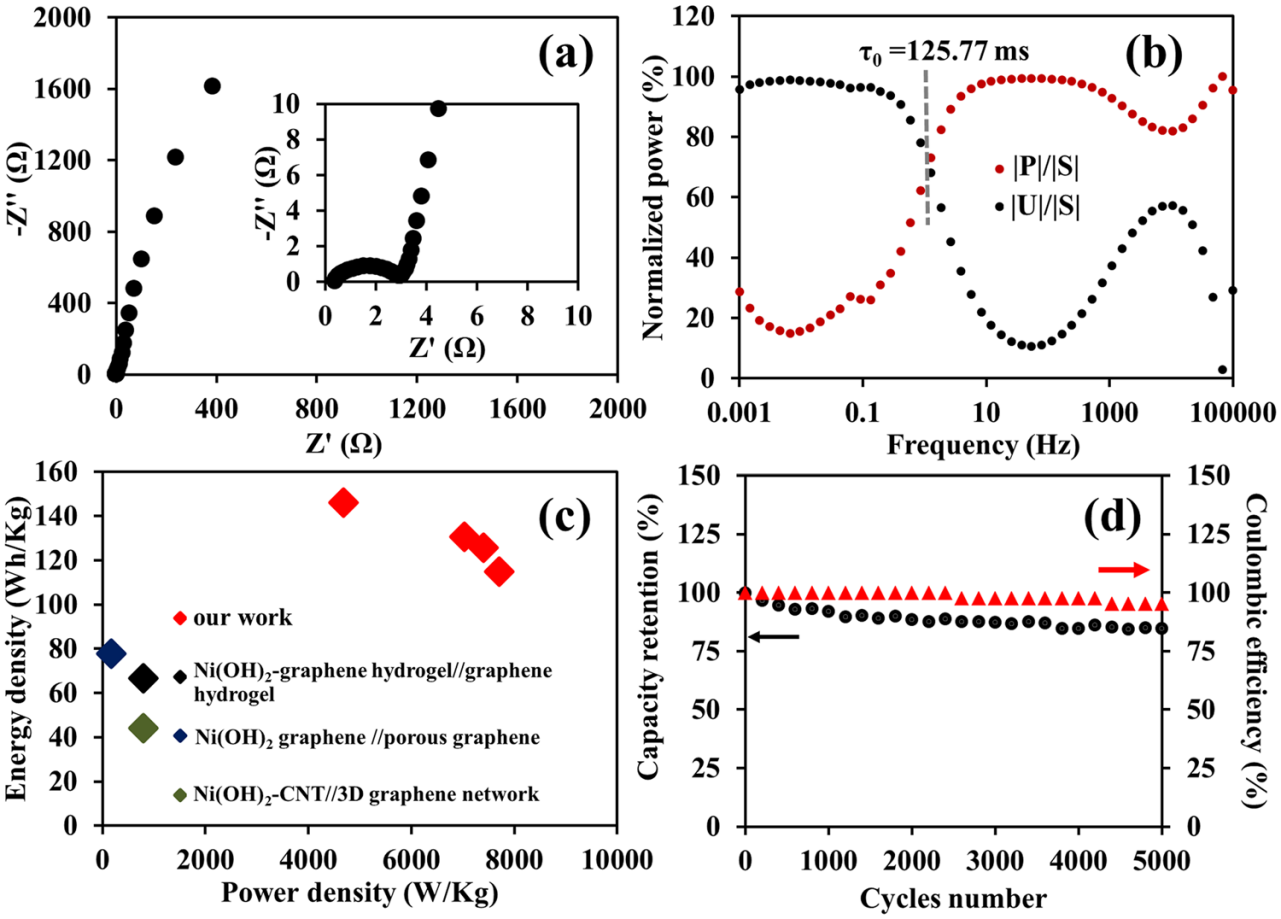
The electrochemical properties of the Ni(OH)_2_-N-rGO_ae_//N-rGO_ae_ AES: (**a**) Nyquist plot, (**b**) the plots of normalized power with the frequency, (**c**) maximum power density vs. energy density compared with some previous report^34, 43, 44^ and (**d**) the capacity retention over 5000 cycles at 1 A g^−1^.

The electrochemical impedance spectroscopy (EIS) of the as-fabricated AES was eventually performed by applying a sinusoidal signal of 10 mV amplitude from 1 mHz to 100 kHz. The bottom left-hand corner of the Nyquist plot in Fig. 5a shows a high frequency result for which 2.6 Ω of the charge transfer resistance (*R*_*ct*_) due to the redox reaction and 0.4 Ω of the internal resistance (*R*_*s*_) are observed. Note, the *R*_*s*_ here is rather small when compared with the other previous work^35^. To further investigate the power efficiency of the as-fabricated AES, the relaxation time (τ_0_), which is the minimum time required for discharging the stored charges from the AES, was determined and shown in Fig. 5b. The smaller τ_0_ represents the higher power efficiency of the AES^36^. The response of frequency (*f*_0_), which is corresponding to the maximum point energy curve, is estimated to be at 1.26 Hz. The determined τ_0_, which is equal to 1/2 *π*f_0_, is 125.77 ms indicating that the as-fabricated AES can provide a quick discharging process and also substantially faster than other previous report^35^. Remarkably, the device can display a high specific energy of 146 Wh kg^−1^ at a specific power of 4687 W kg^−1^ with the remaining of 115 Wh Kg^−1^ at 7705 W kg^−1^ (Fig. 5c). The highest specific energy here is much greater than 77.8 Wh kg^−1^ of the asymmetric supercapacitor of the Ni(OH)_2_-graphene//graphene^4^ and significantly higher than 35.7 Wh kg^−1^ of the amorphous Ni(OH)_2_ supercapacitor^6^. The as-fabricated AES provides high capacity retention over 84.61% after 5000 cycles (see Fig. 5d). This number is rather high when compared with NiCd and NiMH batteries and other Ni(OH)_2_-based supercapacitors^6^. As the result, the Ni(OH)_2_-N-rGO_ae_//N-rGO_ae_ is ideally suitable for many high power and energy applications.

### *Ex situ* evaluation of the AES device

**Figure 6:**
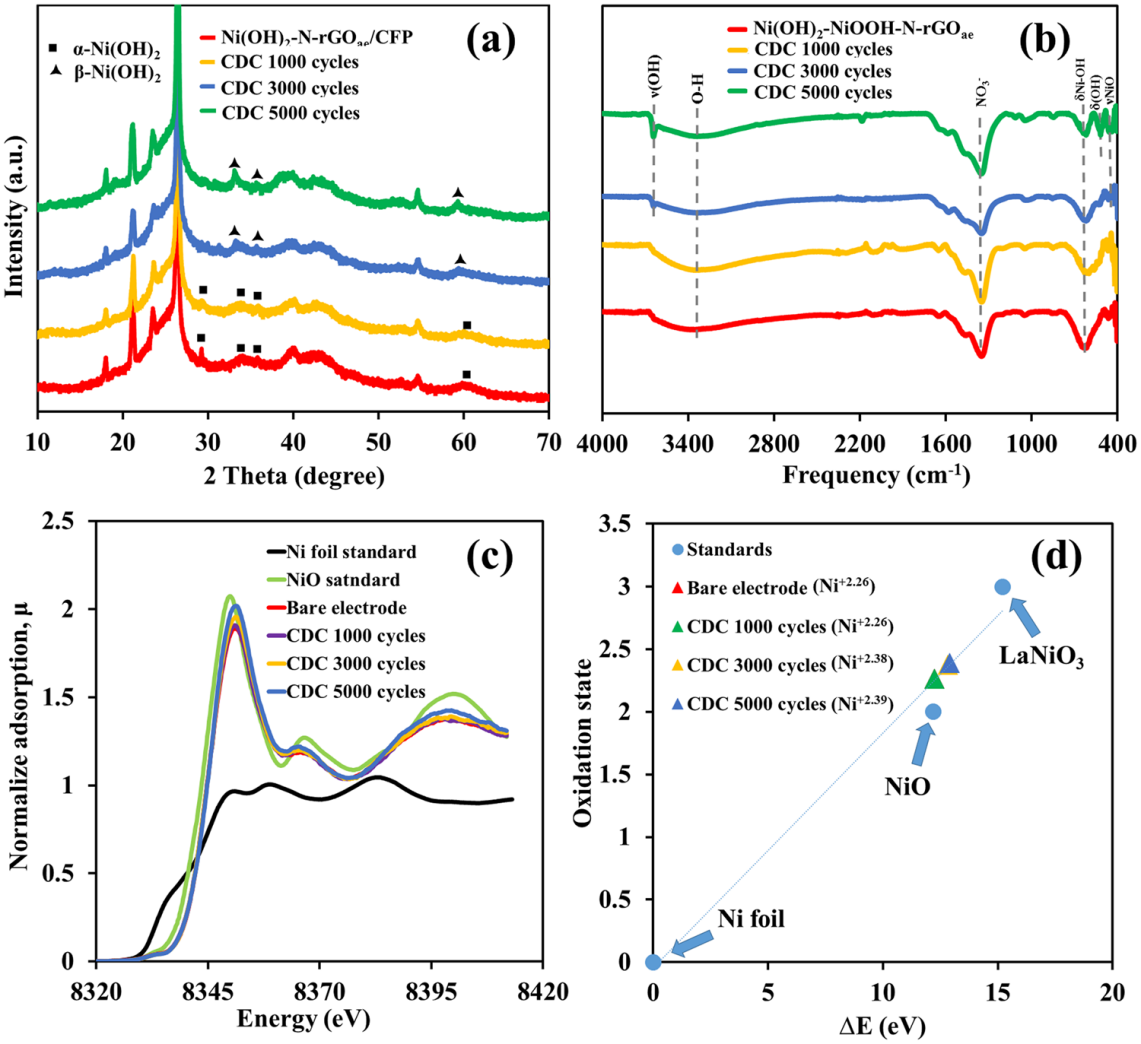
(**a**) XRD patterns and (**b**) FTIR spectra of the Ni(OH)_2_-N-rGO_ae_/CFP electrodes after charged/discharged as well as (**c**) *ex situ* high-resolution XAS spectra and (**d**) the oxidation states vs. ΔE (eV) of the Ni(OH)_2_-N-rGO_ae_ after 1000, 3000 and 5000 cycles as compared with the Ni standard compounds.

After the AES devices were electrochemically tested, *ex situ* measurements of the disassembled positive electrodes were carried out by XRD, FTIR, XAS and XPS. The XRD results in Fig. 6a can imply that α-Ni(OH)_2_ on the positive electrode after tested begins to convert to β-Ni(OH)_2_ after 3000 cycles. Additionally, XRD patterns of β-Ni(OH)_2_ with the diffraction peaks (100), (101), and (110), are initially witnessed on the XRD patterns of the Ni(OH)_2_-N-rGO_ae_ after 3000 cycles. The calculated interlayer spacing is reduced from 7.00 Å (α-Ni(OH)_2_, JCPDS 38–715) to 4.45 Å (β-Ni(OH)_2_, JCPDS 14–0117). The less interlayer spacing can obstruct the diffusion of the OH^−^(H_2_O)_*n*_ (where *n* ≥ 1) clusters to the Ni(OH)_2_ layers. To further investigate the phase transformation of α-Ni(OH)_2_ to β -Ni(OH)_2_ after long cycling, the Ni(OH)_2_-N-rGO_ae_ electrodes after tested at 1000, 3000 and 5000 cycles were investigated by FTIR and XPS techniques (see FTIR in Fig. 6b and XPS in Figure S5). In addition, *ex situ* XAS was applied to investigate the oxidation number of Ni element in the positive electrodes of the AESs after 1000, 3000 and 5000 cycles. The XANES spectra and oxidation state for each sample are shown in Fig. 6c and d, respectively. The Ni K-edge XANES of the samples was recorded and compared with Ni foil (Ni^+0^, 8333 eV), NiO (Ni^+2^, 8345.20 eV) and LaNiO_3_ (Ni^3+^, 8348.20 eV) standards^30^. The adsorption edge energy of Ni on the electrode and after tested for 1000, 3000, 5000 cycles are 8345.25 eV (Ni^+2.26^), 8345.84 eV (Ni^+2.38^) and 8345.91 eV (Ni^+2.39^), respectively. These results indicate that the Ni(OH)_2_ phase begins to transform after 3000 cycles. After 5000 cycles, the phase of α-Ni(OH)_2_ is completely changed to β-Ni(OH)_2_. When the interlayer distance is decreased, the charge mechanism does no longer follow the reversible reaction (1) anymore, the β-Ni(OH)_2_ phase will get oxidized and turned to β-NiOOH rather than *γ*-NiOOH (see Figure S6) leading to poor capacity retention typically detected in the α-Ni(OH)_2_-based AESs. The reaction mechanism is shown in details in Figure S6 of the supporting information.

## Conclusions

N-rGO_ae_ was produced from GO through a hydrothermal reduction with hydrazine and coated on the CFP via a spray-coating technique. α-Ni(OH)_2_ was coated on the N-rGO_ae_ substrate by an electrodeposition using a chronoamperometry at −0.9 V vs Ag/AgCl for 5 min. The charge storage mechanism of α-Ni(OH)_2_-coated N-rGO_ae_ was investigated by *in situ* EQCM and XAS. The mass change of the positive electrode during the charging/discharging processes determined by the EQCM is about 8.5 µg cm^−2^ owing to the insertion/desertion of solvated OH^−^ ions into the interlayer spacing of α-Ni(OH)_2_. At the negative electrode, the EQCM shows that the mass change of the solvated K^+^ electrolytes, physically adsorbed/desorbed to the N-rGO_ae_ electrode via the EDLC mechanism, is 7.5 μg cm^−2^ during the charging/discharging processes. The Ni oxidation state of the Ni(OH)_2_-N-rGO_ae_ electrode after charged at 0.0 V vs. Ag/AgCl is +2.26 and remained stable at this oxidation state until 0.35 V vs. Ag/AgCl before oxidized to +2.76 and +2.79 after charged at 0.5 and 0.6 V vs. Ag/AgCl, respectively. This is due to the oxidation reaction of Ni(OH)_2_ with OH^−^ providing NiOOH. For the discharging process, the Ni oxidation state of the Ni(OH)_2_-N-rGO_ae_ electrode is reduced back to +2.76 after discharged at 0.4 V vs. Ag/AgCl and +2.26 at 0 V vs Ag/AgCl. These results show good reversible redox reaction of the Ni(OH)_2_-N-rGO_ae_ electrode. A single coin-cell AES of Ni(OH)_2_-N-rGO_ae_//N-rGO_ae_ (CR2016) was assembled using a hydrolyzed polyethylene separator absorbed with 1 M KOH. The as-fabricated energy storage can provide a wide working voltage of 1.5 V with a high specific energy of 146 Wh kg^−1^ and a maximum specific power of 7705 W kg^−1^ with 84.61% capacity retention after 5000 cycles. Interestingly, after 3000 cycles, the α-Ni(OH)_2_, which has a wide interlayer separation of 7.0 Å, was partially changed to β-Ni(OH)_2_, which has a narrower interlayer separation of 4.45 Å. This leads to the capacity retention decay since the β-Ni(OH)_2_ has too narrow interlayer spacing for the solvated electrolytes, OH^−^(H_2_O)n where n ≥ 1. This energy storage device has a potential to be used in high energy and power applications. Also, this device has a bright potential to replace the NiCd and NiMH technologies since we do not need toxic Cd and expensive La alloy at the negative electrode.

## Experimental Section

### Synthesis of nitrogen-doped reduced graphene oxide (N-rGO_ae_)

A hydrothermal reduction method was used to synthesize N-rGO_ae_. Briefly, 3 mg/ml of GO from the modified Hummers method^26, 37–39^ was prepared in Milli-Q water using ultrasonication for 30 min. 0.5 M hydrazine hydrate (N_2_H_4_, 80% Merck) was added to the mixture, and then the suspension was poured into a Teflon-lined autoclave and heated at 100 °C for 3 h. After naturally cooling down the autoclave to room temperature, a vacuum filtration is used to collect the N-rGO hydrogel. The as-filtrated N-rGO hydrogel was left in Milli-Q water at 25 °C and the water was daily replaced for washing out the remaining of reducing agent. After that the hydrogel was frozen at 0 °C for 24 h. Then, the frozen hydrogel was put in a freezing dryer to remove water at −55 °C for 72 h to form the final product N-rGO_ae_. Next, a spray coating technique is applied using an airbrush with 0.3 mm of brush nozzle (Paasche Airbrush Company, USA) to coat the N-rGO_ae_ on the CFP (Carbon fiber paper) surface at 20 psi and room temperature. Finally, the electrode was dried in vacuum oven at 60 °C for 24 h^40^.

### Electrodeposition of nickel hydroxide (α-Ni(OH)_2_) on N-rGO_ae_ electrode

Firstly, α-Ni(OH)_2_ was deposited on the N-rGO_ae_-coated CFP substrate by applying −0.9 V vs. Ag/AgCl for 5 min through a chronoamperometry method using a Metrohm AUTOLAB potentiostat (PGSTAT 302 N made in Netherlands running NOVA version 1.10.3 software). The electrodeposition solution (60 ml) is 0.1 M nickel (II) nitrate hexahydrate (Ni(NO_3_)_2_.6H_2_O, 97% QRec) in 0.5 M sodium nitrate (NaNO_3_, 99.5% QRec). The counter electrode is the platinum wire and the reference electrode is the Ag/AgCl (3 M KCl). After electrodeposition process, the as-electrodeposited α-Ni(OH)_2_ (0.97 mg) on the 0.5-mg N-rGO_ae_-coated CFP substrate was rinsed 5 times using Milli-Q water before putting into a vacuum dryer at 50 °C overnight to dry off the samples.

### Material characterizations

Field-emission scanning electron microscopy (FE-SEM, JSM-7001F (JEOL Ltd., Japan)) and transmission electron microscopy (TEM, JEM 1220 (JEOL Ltd., Japan)) were applied to study the morphologies of the as-prepared materials. The structures of the materials were examined by X-ray diffraction (XRD, Bruker optics, Germany) using a monochromatic Cu Kα radiation (λ = 0.15405 nm), Fourier Transform Infrared Spectrometer (FT-IR, PerkinElmer Paragon 1000) and Raman spectroscopy (Senterra Dispersive Raman Microscope, Bruker) with an excitation wavelength of 532 nm. The chemical composition on the surface of the materials was characterized by X-ray photoelectron spectroscopy (XPS, Axis Ultra DLD, Kratos Analytical Ltd) with Al-K alpha radiation (hʋ = 14,866 eV).

For X-ray absorption spectroscopy (XAS) measurement, Ni K-edge fluorescent XAS was performed at a beamline No. 5.2 at the Synchrotron Light Research Institute (Public Organization), Nakhon Ratchasima, Thailand. The Ge(220) double-crystal monochromator (energy range 3440–12100 eV) is used and a fluorescence mode with a 4-element silicon drift detector, which was placed 90° to the beam and 45° to the sample, is applied to record the spectroscopic data. The Ni K-edge (8333 eV) was calibrated using the Ni foil before measurement. The light dimension on the sample was adjusted to 5 mm width and 1 mm height.

The charge storage mechanism of Ni(OH)_2_-N-rGO_ae_ electrode was studied through the *in situ* XAS technique together with the chronoamperometry in 1 M KOH electrolyte by applying the potentials stepped from 0.0, 0.35, 0.5 to 0.6 V vs. Ag/AgCl following by the backward process from 0.6, 0.4, 0.18 to 0 V vs. Ag/AgCl. For reaching the steady state, each step potential was hold for 15 min before starting the XAS measurement^41, 42^. Additionally, acrylic sheets, which are used as the materials of the electrochemical cell, has a dimension of 2 × 2 × 3.5 cm^3^ with a drilled hole diameter of 1 cm on one 2-cm^2^ side of the acrylic sheet. The drilled hole was covered by a larger piece of polypropylene film. During the process for measuring, the Ag/AgCl in 3 M KCl (reference electrode) and Pt wire (counter electrode) were located near the Ni(OH)_2_-N-rGO_ae_ electrode at a distance of ca.1 cm. The *ex situ* Ni K-edge XAS was also carried out in the fluorescence mode.

### Fabrication of AES and the electrochemical evaluation

The AES was assembled of the negative and positive electrodes with a geometrical coin cell (CR2016). Hydrolyzed polyethylene (PE) film with a thickness of 25 μm, which was used as the separator, was soaked in 1 M KOH for 10 min before used. Then, the separator was inserted between the positive electrode (1.47-mg Ni(OH)_2_-N-rGO_ae_ coated on CFP) and the negative electrode (2.53-mg N-rGO_ae_ coated on CFP). Finally, the coin cell was then assembled by pressing with a crimper machine at 100 psi. The charge storage mechanism was performed by the EQCM technique via a cyclic voltammetry (CV) combining with quartz crystal microbalance techniques of Au/TiO_2_. The EQCM electrode was tested in atmospheric pressure and at room temperature (25 °C) using a three-electrode system; Ag/AgCl (3 M KCl in gel) as a reference electrode and Au spiral shape as a counter electrode. The electrochemical property of the as-fabricated supercapacitors was evaluated by CV, galvanostatic charge–discharge (GCD), and electrochemical impedance spectroscopy (EIS) using a Metrohm AUTOLAB potentiostat (PGSTAT 302 N) made in Netherlands running NOVA software (version 1.11).

## Electronic supplementary material

Supporting Information
