# Supplementary material for: Hybrid Energy Storage of Ni(OH)2-coated N-doped Graphene Aerogel//N-doped Graphene Aerogel for the Replacement of NiCd and NiMH Batteries
Source: Sci Rep. 2017 Apr 25;7:1124. doi: 10.1038/s41598-017-01191-8 (PMC5430751; doi:10.1038/s41598-017-01191-8)
Supplement: Supplementary file 1 — Supporting Information [file 41598_2017_1191_MOESM1_ESM.pdf]

Supporting Information

Hybrid Energy Storage of $Ni(OH)_2$-coated N-doped Graphene Aerogel//N-doped Graphene Aerogel for the Replacement of NiCd and NiMH Batteries

Pichamon Sirisinudomkit[1,2], Pawin Iamprasertkun[1], Atiweena Krittayavathananon[1], Tanut Pettong[1], Peerapan Dittanet[2], and Montree Sawangphruk[1,*]

[1]Department of Chemical and Biomolecular Engineering, School of Energy Science and Engineering, Vidyasirimedhi Institute of Science and Technology, Rayong 21210, Thailand
[2]Department of Chemical Engineering, Centre for Advanced Studies in Nanotechnology and Its Applications in Chemical Food and Agricultural Industries, and NANOTEC-KU-Centre of Excellence on Nanoscale Materials Design for Green Nanotechnology, Kasetsart University, Bangkok 10900, Thailand

1. Calculation

The mass change (Δm) determined by an EQCM technique

The measured quartz resonance frequency change (Δf) can be converted in to mass change (Δm) according to the derived Sauerbrey equation (s1)[1] below;

$$\Delta f = -\Delta m C_f \qquad \text{(s1)}$$

where the frequency (Δf) in Hz and the calibration constant (C_f) is 0.0815 Hz $ng^{-1}cm^2$.

Optimization of the mass ratio between positive and negative electrodes

The equation (s2) below is applied to determine the optimized masses of positive and negative electrodes;

$$\frac{m_+}{m_-} = \frac{C_{s-}\Delta V_-}{C_{s+}\Delta V_+} \qquad \text{(s2)}$$

where m is the mass of active material, C_s is the specific capacitance, and ΔV is the voltage range for positive(+) and negative(–) electrodes[2,3]. According to the CV test at 25 mV s^{-1}, the mass ratio between α-$Ni(OH)_2$-N-rGO_{ae} and N-rGO_{ae} is 0.58.

$$\frac{m_{+}}{m_{-}} = \frac{(403.46)(0.9)}{(516.95)(1.2)} = 0.58$$

In this experiment, the active material is 4.0 mg which are from 1.47 mg of $Ni(OH)_2$-$NrGO_{ae}$ on positive electrode and 2.53 mg of N-rGO_{ae} on negative electrode.

The specific capacitance of the supercapacitors

The specific capacitance ($C_{cell,\ cv}$) of the supercapacitor cell can be determined from the CV technique by the following equation (s3);

$$C_{cell,cv} = \frac{Q}{\Delta V_{cv} \times m} \tag{s3}$$

where Q is an average charge in the discharge process (Coulomb) of the CV curve, ΔV_{cv} is a working potential window (V) and m is a total mass of active material used in both positive and negative electrodes.

The specific capacitance ($C_{cell,\ GCD}$) of the supercapacitor cell can also be determined from the galvanostatic charge discharge (GCD) technique by the following equation (s4);

$$C_{cell,GCD} = \frac{I_{GCD} \times \Delta t_{GCD}}{\Delta V_{GCD} \times m} \tag{s4}$$

where I_{GCD} is the applied constant current (A), Δt_{GCD} is the discharge time (s) and ΔV_{GCD} is the working potential window (V) of the cell excluding the *iR* drop.

b values

The voltammetric response at various scan rates from 10 to 100 mV s^{-1} was calculated by following the equation (s5) below;

$$I = av^{b} \tag{s5}$$

where *I* is a current, v is the scan rate. *a* and *b* are adjustable parameters for which *b* is determined from the slope of the linear plot of *log I* versus *log v*. In general, *b* is equal to 1 involving non-diffusion controlled capacitive effect while b is equal to 0.5 indicating the ideal diffusion-controlled redox intercalation process (battery-type behavior).

Specific energy and maximum specific power of the supercapacitor cells

Specific energy (E_{cell}) and maximum specific power (P_{max}) will be calculated from the following equation;

$$E_{cell} = \frac{1}{2} C_{cell} \Delta V^2 \quad (s6)$$

$$P_{max} = \frac{V_0^2}{4R_{cell}} \quad (s7)$$

2. FESEM images of carbon fiber paper (CFP) and $Ni(OH)_2$-coated CFP

Figure S1a shows an FE-SEM image of CFP for which the average of the carbon fibers is about 8 μm having a PVDF binder on their surfaces. Whilst, Figure S1b shows an FE-SEM image of the as-electrodeposited α-$Ni(OH)_2$ on the CFP.

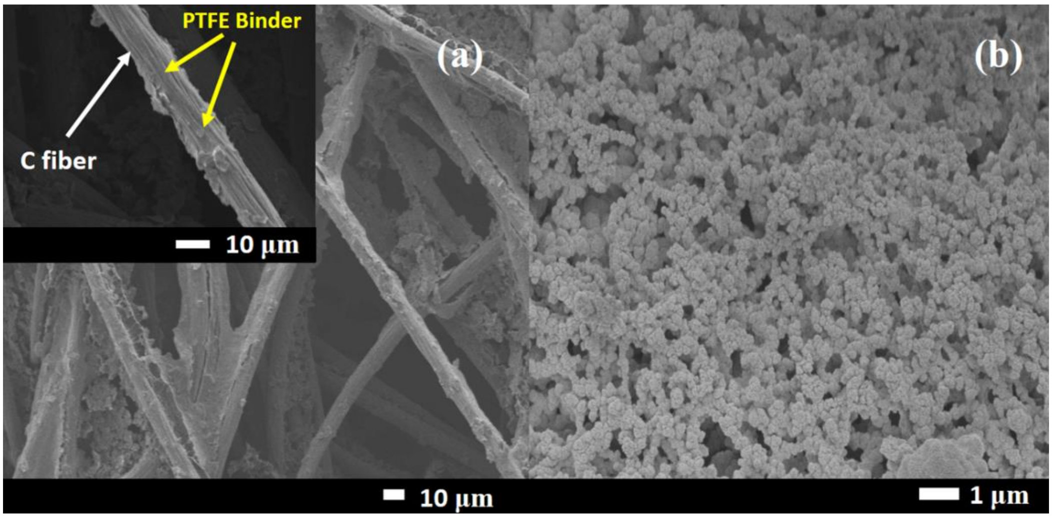

Figure S1. FE-SEM images of (a) CFP and (b) the as-electrodeposited α-$Ni(OH)_2$ on CFP.

Figure S2 shows FE-SEM image as well as the corresponding EDS elemental mapping images of carbon, nickel and oxygen of the $Ni(OH)_2$-N-rGO$_{ae}$/CFP.

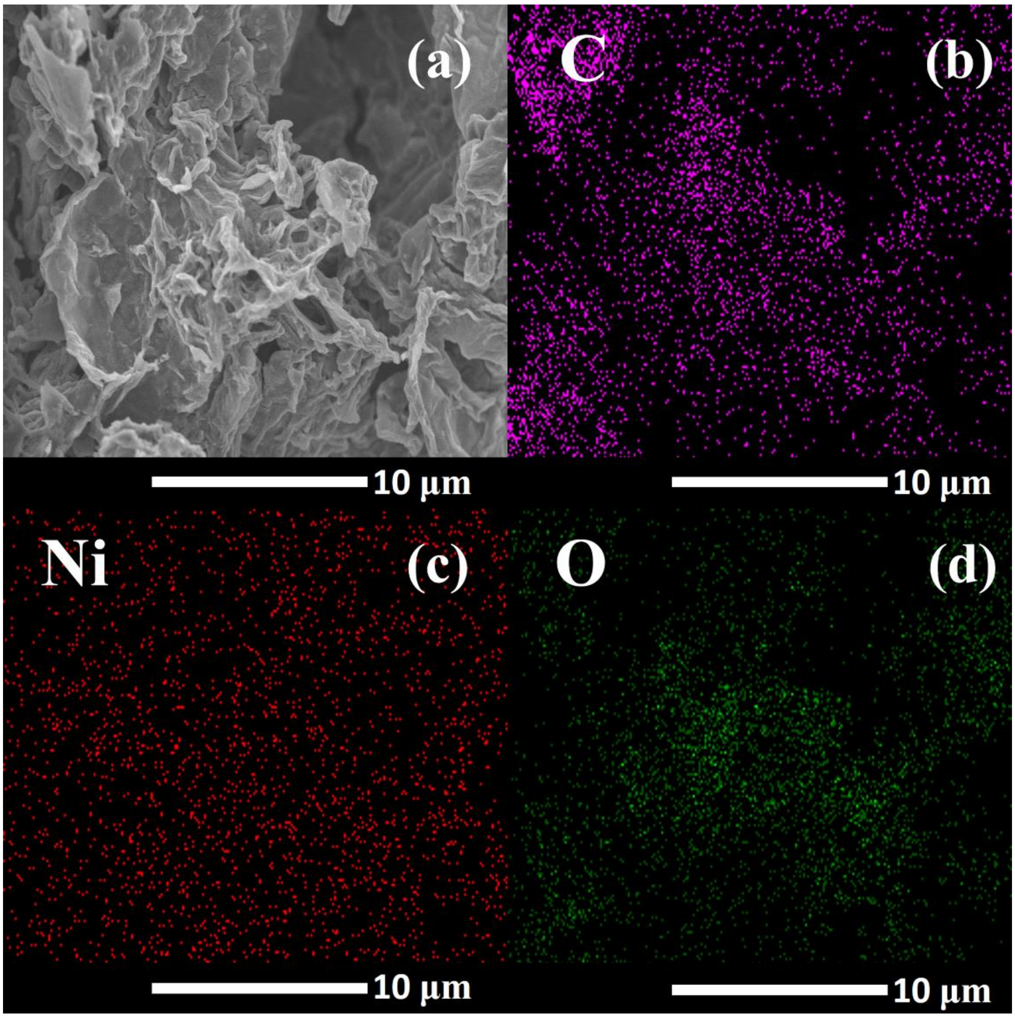

Figure S2. (a) FE-SEM image as well as the corresponding EDS elemental mapping images of (b) carbon, (c) nickel and (d) oxygen of the $Ni(OH)_2$-N-rGO_{ae}.

3. XRD and Raman of $Ni(OH)_2$, N-rGO_{ae} and $Ni(OH)_2$-N-rGO_{ae} coated CFPs

The crystalline structure of the as-prepared materials was characterized by XRD and Raman techniques. The as-synthesized N-rGO_{ae} in Figure S3a displays the (002) diffraction plane as a broad peak at 25.5°, which presents a small domain of graphitic layers. Besides, a broad peak at 43.3° corresponding to the (100) plane indicates a honeycomb structure of sp^3 carbon atoms linked with oxygen- and nitrogen-containing functional groups[4]. From Figure 3a, $Ni(OH)_2$ and $Ni(OH)_2$-N-rGO_{ae} exhibit the same XRD characteristics of rhombohedral α-$Ni(OH)_2$ structure (JCPDS 38-715)[5] with the lattice parameters of a = b = 3.08 Å and c = 23.41 Å. The calculated interlayer distance is found to be equal to the interlayer distance of previous report ca. 7.0 Å [6]. Additionally, the crystallite size of $Ni(OH)_2$ on N-rGO_{ae} calculated by using Sherrer's equation

is ca. 22.32 nm. For the diffraction peaks around 22°, 32° and 53°, they are the characteristic peaks of α -$Ni(OH)_2$-NiOOH (JCPDS 006-0044)[7].

The Raman spectra of both $Ni(OH)_2$ and $Ni(OH)_2$-N-rGO_{ae} in Figure S2b performed display two peaks at 462 and 563 cm^{-1} because of Ni-O and Ni-OH stretching from α-$Ni(OH)_2$, respectively. The vibrational mode of the O–H bond from hydroxyl groups is presented as the peak at 3650 cm^{-1}. Besides, the vibrational modes of the nitrate ions (NO_3^-) are at 719 (symmetric stretching mode), 1049 (in-plane bending mode) and 1362 cm^{-1} (fingerprint of inter-layer NO_3^- ions), respectively[8,9]. Whilst, two distinct bands of the N-rGO_{ae} and $Ni(OH)_2$-N-rGO_{ae} at 1595 and 1350 cm^{-1} are owing to G and D bands of N-rGO_{ae}, respectively[10]. A ratio of disordered and ordered contents of the N-rGO_{ae} sheets (I_D/I_G) calculated is 0.91.

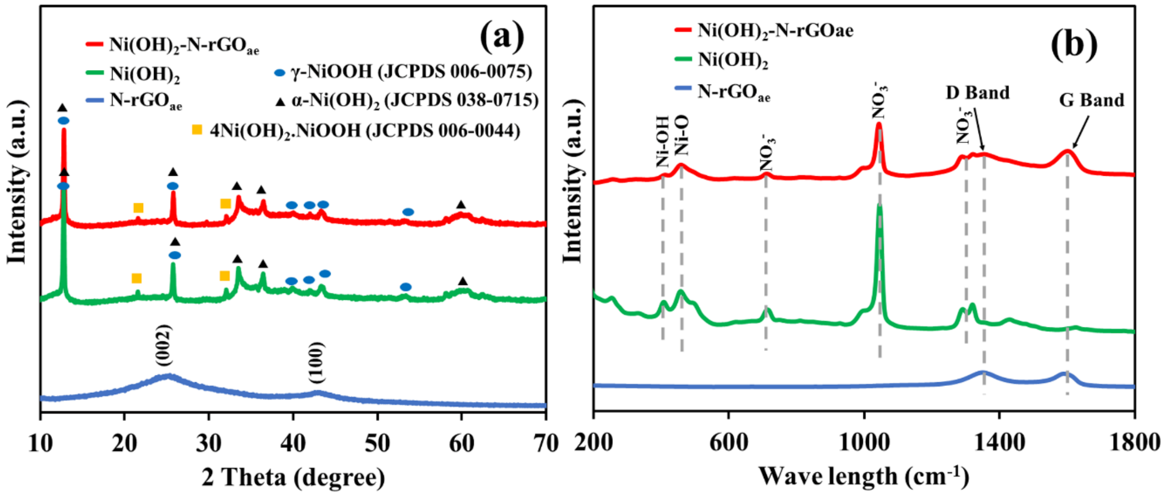

Figure S3. (a) XRD patterns and (b) Raman spectra of $Ni(OH)_2$, N-rGO_{ae} and $Ni(OH)_2$-N-rGO_{ae}.

4. XPS spectra of N-rGO_{ae} and $Ni(OH)_2$- N-rGO_{ae}

X-ray photoelectron spectroscopy (XPS) is used to investigate the chemical composition of $Ni(OH)_2$ -N-rGO_{ae} on CFP. In Figure S4a, a wide scan of the XPS spectra of N-rGO_{ae} on CFP mainly contains C_{1s}, O_{1s} and N_{1s} peaks. Whilst, the XPS spectra of $Ni(OH)_2$-N-rGO_{ae} on CFP composes of C_{1s}, Ni_{2p} and O_{1s} core level peaks. In addition, sulfur peaks are observed on their nanoscale surfaces due to an adhesive used in the production process of CFP[11]. For the C_{1s} region (Figure S4b), the spectrum shows bonding types at 284.6, 285.0, 285.7, 286.7, 288.8 and 289.6 eV corresponding to the C-C, C=C, C-OH/ C-O-C, O=C-OH and CF_2, respectively

referring to N-rGO$_{ae}$ and CFP[11,12]. In addition, Ni 2p spectrum consists of Ni 2p$_{3/2}$ core level peaks at 855.6 eV and 857.3 eV with their satellite peaks at 861.24 and 863.25 eV, respectively. Ni 2p$_{1/2}$ peaks at 872.77 eV and 874.52 eV with their satellite peaks at 878.11 and 880.10 eV, respectively (Figure S4c). This confirms the characteristics of α-Ni(OH)$_2$[13] and γ-NiOOH[14]. For O1s region, the peak at 530.7 eV is assigned to C-O-Ni bond. The peak at 531.8 eV is demonstrated to C=O groups or shoulder peak of O1s in Ni(OH)$_2$. The peaks of C-OH and C-O-C groups are presented at 532.8 eV (Figure S4d)[8,15].

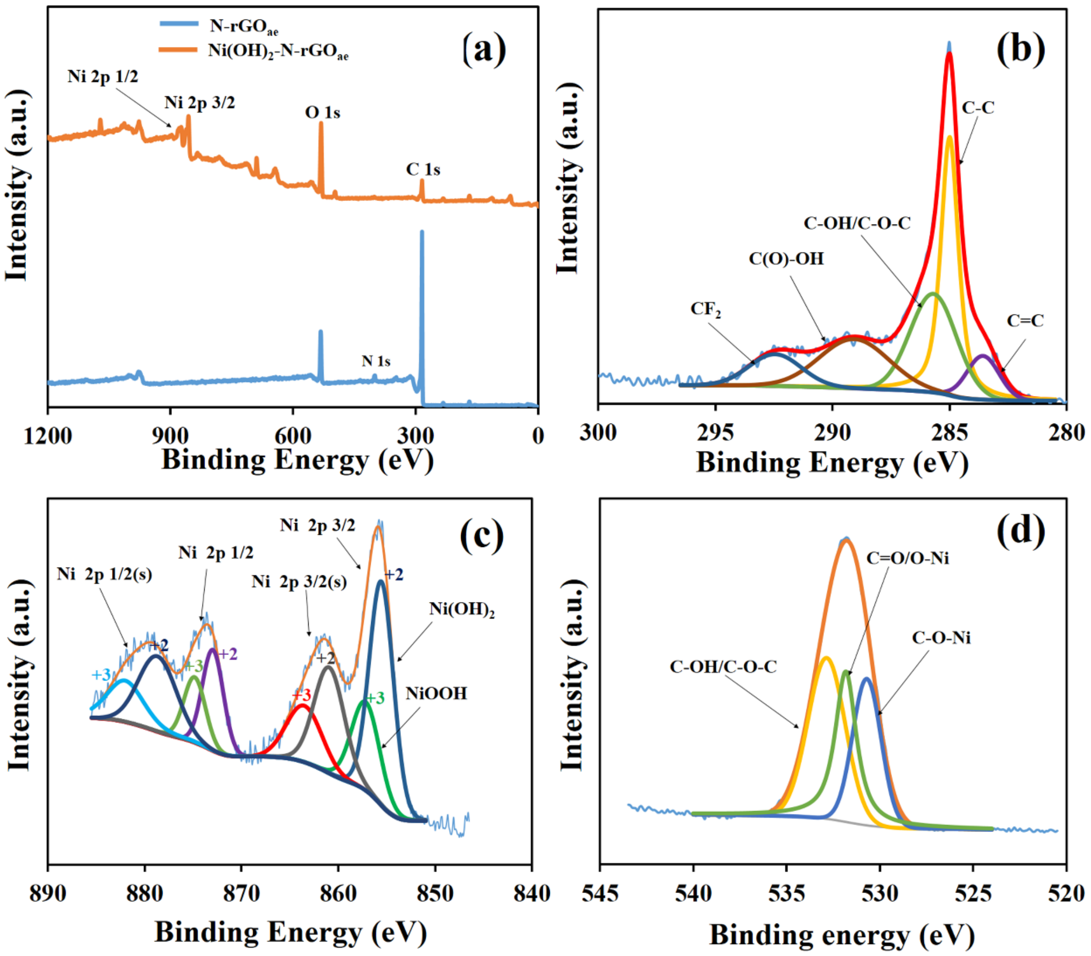

Figure S4. (a) The survey XPS spectrum of N-rGO$_{ae}$ and Ni(OH)$_2$-N-rGO$_{ae}$. (b) C 1s spectrum, (c) Ni 2p spectrum and (d) O 1s spectrum of Ni(OH)$_2$-N-rGO$_{ae}$.

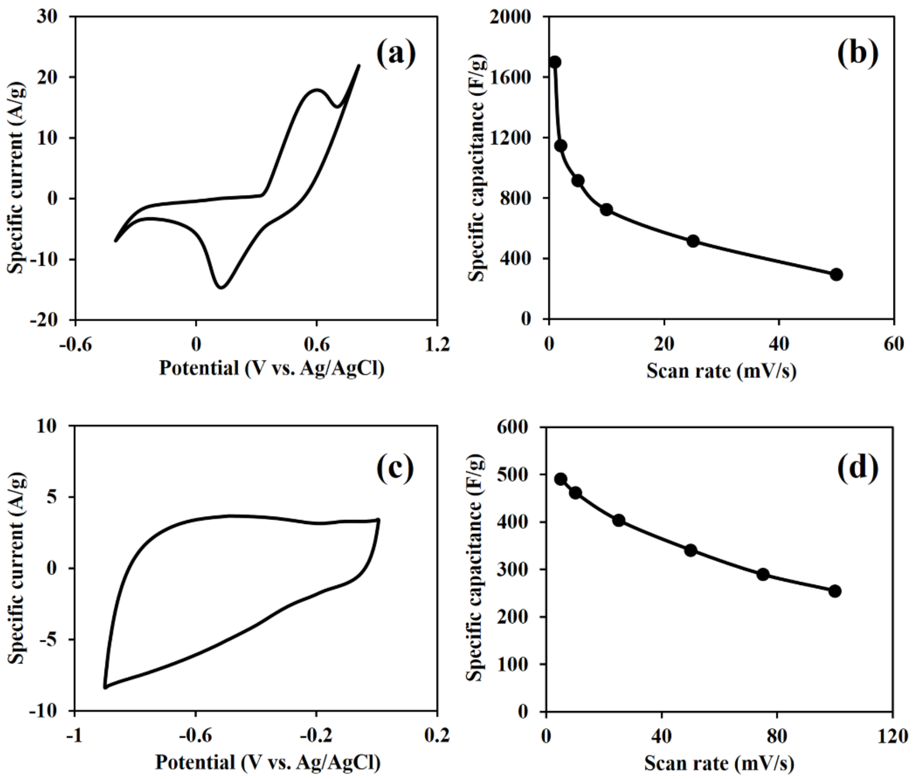

Figure S5. (a) CV curve and (b) specific capacitance as a function of the scan rates of the $Ni(OH)_2$-N-rGO$_{ae}$ electrode/CFP at 10 mV s^{-1} in 1 M KOH as well as (c) CV curve and (d) specific capacitance as a function of the scan rates of the N-rGO$_{ae}$ electrode at 10 mV s^{-1} in 1 M KOH.

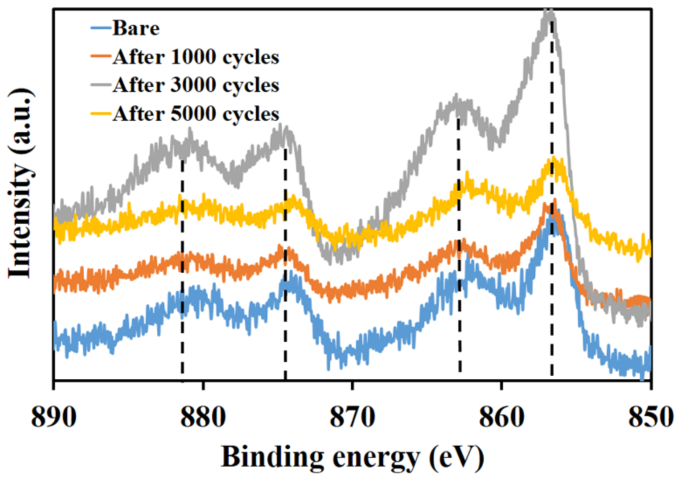

Figure S6. (a) Ni 2p spectrum of the $Ni(OH)_2$-N-rGO$_{ae}$ electrodes after charged/discharged.

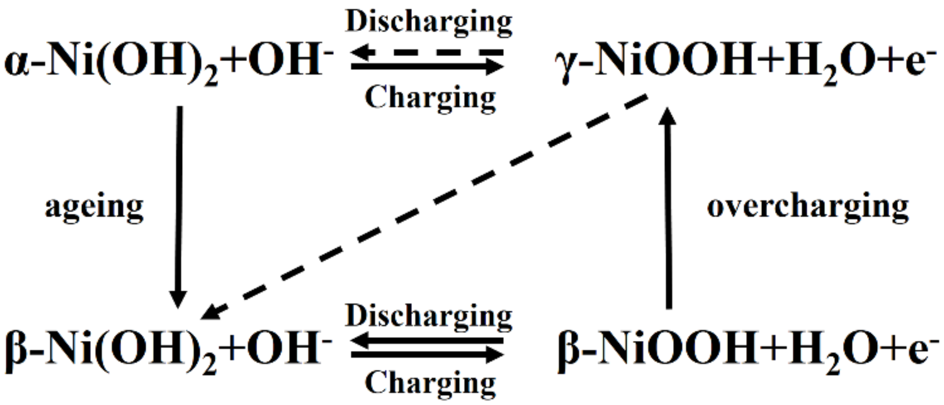

Figure S7. A schematic of the electrochemical processes that occur at the nickel hydroxide positive electrode**.**

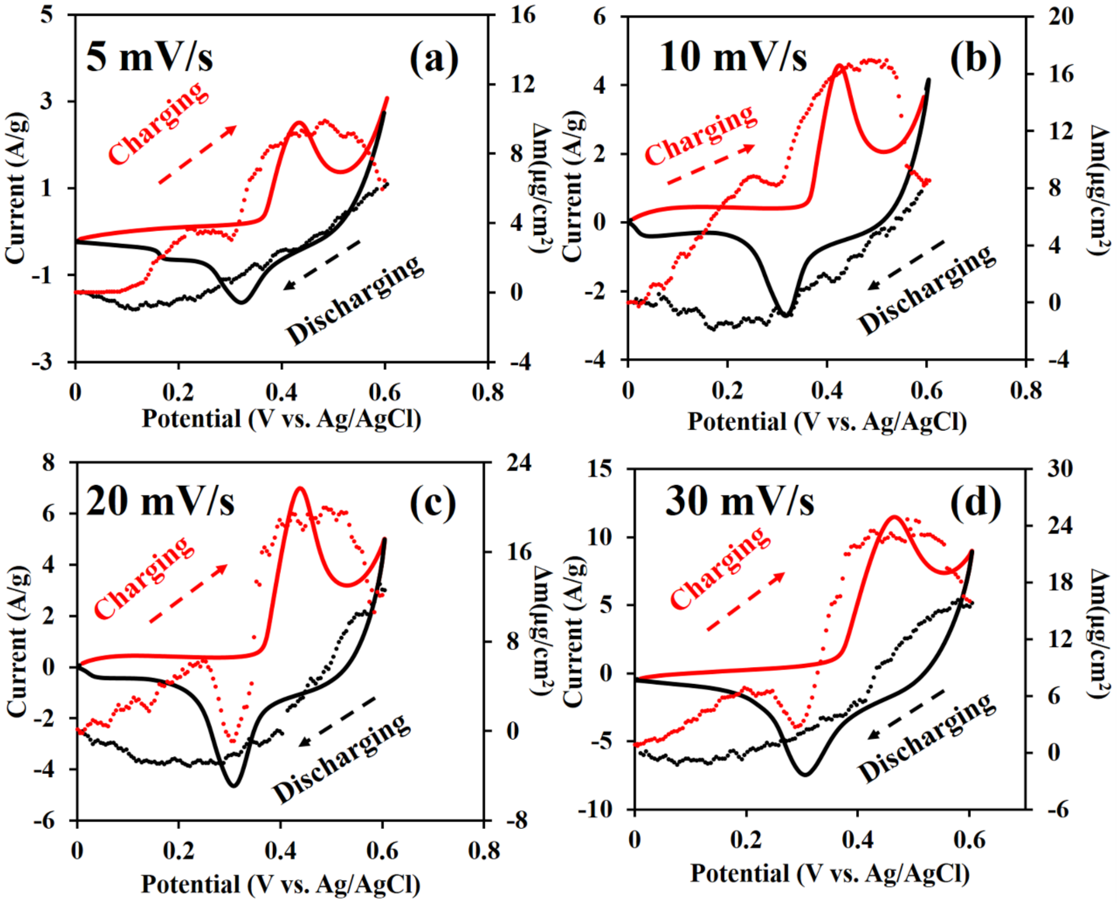

Figure S8. EQCM responses and CVs of α-$Ni(OH)_2$-N-rGO$_{ae}$ at different scan rates.

Table S1. The charge storage performances of the Ni-based asymmetric supercapacitors.

Materials	Electrolytes	Testing methods	Capacitances	Refs.
β-$Ni(OH)_2$ on Ni foam//activated carbon	6 M KOH	GCD at 0.13 A g^{-1}	105.8 F g^{-1}	[16]
$Ni(OH)_2$ // activated carbon	1 M KOH	CV at 5 mV s^{-1}	153 F g^{-1}	[17]
$NiCo_2O4$-reduced graphite oxide//activated carbon	2 M KOH	GCD at 0.5 A g^{-1}	99.4 F g^{-1}	[18]
Ni-Co oxide// activated polyaniline-derived carbon	2 M KOH	GCD at 0.5 A g^{-1}	202 F g^{-1}	[19]
CNT@$Ni(OH)_2$// 3D graphene network	1 M KOH	GCD at 1 A g^{-1}	123.7 F g^{-1}	[20]
$Ni(OH)_2$-graphene//porous graphene	6 M KOH	CV at 5 mV s^{-1}	218.4 F g^{-1}	[21]
$Ni(OH)_2$-N-rGO_{ae}//N-rGO_{ae}	1 M KOH	GCD at 1 A g^{-1}	487.1 F g^{-1}	This work

REFERENCES

1 Sauerbrey, G. Verwendung von Schwingquarzen zur Wägung dünner Schichten und zur Mikrowägung. *Z. Phys.* **155**, 206-222 (1959).

2 Wang, R., Xu, C. & Lee, J.-M. High performance asymmetric supercapacitors: New NiOOH nanosheet/graphene hydrogels and pure graphene hydrogels. *Nano Energy* **19**, 210-221 (2016).

3 Lin, T.-W., Dai, C.-S. & Hung, K.-C. High Energy Density Asymmetric Supercapacitor Based on NiOOH/Ni3S2/3D Graphene and Fe3O4/Graphene Composite Electrodes. *Sci. Rep.* **4**, 7274, doi:10.1038/srep07274 (2014)

4 Sahu, V. *et al.* Heavily nitrogen doped, graphene supercapacitor from silk cocoon. *Electrochim. Acta* **160**, 244-253 (2015).

5 Yan, J. *et al.* Advanced asymmetric supercapacitors based on Ni(OH) 2/graphene and porous graphene electrodes with high energy density. *Adv. Funct. Mater.* **22**, 2632-2641 (2012).

6 Jeevanandam, P., Koltypin, Y. & Gedanken, A. Synthesis of Nanosized α-Nickel Hydroxide by a Sonochemical Method. *Nano Lett.* **1**, 263-266 (2001).

7 Alhebshi, N. A., Rakhi, R. B. & Alshareef, H. N. Conformal coating of Ni(OH)2 nanoflakes on carbon fibers by chemical bath deposition for efficient supercapacitor electrodes. *J. Mater. Chem. A* **1**, 14897-14903 (2013).

8 Su, Y.-Z., Xiao, K., Li, N., Liu, Z.-Q. & Qiao, S.-Z. Amorphous Ni(OH)2 @ three-dimensional Ni core-shell nanostructures for high capacitance pseudocapacitors and asymmetric supercapacitors. *J. Mater. Chem. A* **2**, 13845-13853 (2014).

9 Yu, J.-Y., Zhang, Y., Tan, S.-H., Liu, Y. & Zhang, Y.-H. Observation on the Ion Association Equilibria in NaNO3 Droplets Using Micro-Raman Spectroscopy. *J. Phys. Chem.B* **116**, 12581-12589 (2012).

10 Iamprasertkun, P., Krittayavathananon, A. & Sawangphruk, M. N-doped reduced graphene oxide aerogel coated on carboxyl-modified carbon fiber paper for high-performance ionic-liquid supercapacitors. *Carbon* **102**, 455-461 (2016).

11 Suktha, P. *et al.* High-Performance Supercapacitor of Functionalized Carbon Fiber Paper with High Surface Ionic and Bulk Electronic Conductivity: Effect of Organic Functional Groups. *Electrochim. Acta* **176**, 504-513 (2015).

12 Iamprasertkun, P., Krittayavathananon, A. & Sawangphruk, M. N-doped reduced graphene oxide aerogel coated on carboxyl-modified carbon fiber paper for high-performance ionic-liquid supercapacitors. *Carbon* **102**, 455-461 (2016).

13 Zhu, Y. *et al.* Ultrathin Nickel Hydroxide and Oxide Nanosheets: Synthesis, Characterizations and Excellent Supercapacitor Performances. *Sci. Rep.* **4**, 5787, doi:10.1038/srep05787 (2014).

14 Yuan, Y. F. *et al.* Nickel foam-supported porous Ni(OH)2/NiOOH composite film as advanced pseudocapacitor material. *Electrochim. Acta* **56**, 2627-2632 (2011).

15 Wu, Z. *et al.* Electrostatic Induced Stretch Growth of Homogeneous β-Ni(OH)2 on Graphene with Enhanced High-Rate Cycling for Supercapacitors. *Sci. Rep.* **4**, 3669, doi:10.1038/srep03669 (2014).

16 Huang, J. *et al.* Asymmetric supercapacitors based on β-Ni(OH)2 nanosheets and activated carbon with high energy density. *J. Power Sources* **246**, 371-376 (2014).

17 Li, H. B. *et al.* Amorphous nickel hydroxide nanospheres with ultrahigh capacitance and energy density as electrochemical pseudocapacitor materials. *Nat. Commun*. **4**, 1894, doi:10.1038/ncomms2932 (2013).

18 Wang, X., Liu, W. S., Lu, X. & Lee, P. S. Dodecyl sulfate-induced fast faradic process in nickel cobalt oxide-reduced graphite oxide composite material and its application for asymmetric supercapacitor device. *J. Mater. Chem.* **22**, 23114-23119 (2012).

19 Wang, R. & Yan, X. Superior asymmetric supercapacitor based on Ni-Co oxide nanosheets and carbon nanorods. *Sci. Rep.* **4**, 3712, doi:10.1038/srep03712 (2014).

20 Yi, H. *et al.* Advanced asymmetric supercapacitors based on CNT@Ni(OH)2 core-shell composites and 3D graphene networks. *J. Mater. Chem.A* **3**, 19545-19555 (2015).

21 Yan, J. *et al.* Advanced Asymmetric Supercapacitors Based on Ni(OH)2/Graphene and Porous Graphene Electrodes with High Energy Density. *Adv. Funct. Mater.* **22**, 2632-2641 (2012).
